# *Yy1* Gene Dosage Effect and Bi-Allelic Expression of *Peg3*

**DOI:** 10.1371/journal.pone.0119493

**Published:** 2015-03-16

**Authors:** Bambarendage P. U. Perera, Ryoichi Teruyama, Joomyeong Kim

**Affiliations:** Department of Biological Sciences, Louisiana State University, Baton Rouge, Louisiana, 70803, United States of America; University of Bonn, Institut of experimental hematology and transfusion medicine, GERMANY

## Abstract

In the current study, we tested the *in vivo* effects of *Yy1* gene dosage on the *Peg3* imprinted domain with various breeding schemes utilizing two sets of mutant alleles. The results indicated that a half dosage of *Yy1* coincides with the up-regulation of *Peg3* and *Zim1*, suggesting a repressor role of *Yy1* in this imprinted domain. This repressor role of *Yy1* is consistent with the observations derived from previous *in vitro* studies. The current study also provided an unexpected observation that the maternal allele of *Peg3* is also normally expressed, and thus the expression of *Peg3* is bi-allelic in the specific areas of the brain, including the choroid plexus, the PVN (Paraventricular Nucleus) and the SON (Supraoptic Nucleus) of the hypothalamus. The exact roles of the maternal allele of *Peg3* in these cell types are currently unknown, but this new finding confirms the previous prediction that the maternal allele may be functional in specific cell types based on the lethality associated with the homozygotes for several mutant alleles of the *Peg3* locus. Overall, these results confirm the repressor role of *Yy1* in the *Peg3* domain and also provide a new insight regarding the bi-allelic expression of *Peg3* in mouse brain.

## Introduction

*Peg3* (Paternally Expressed Gene 3) was the first imprinted gene identified from the evolutionarily conserved 500-kb domain located in proximal mouse chromosome 7/ human chromosome 19q13.4 [1–3]. Since then, 6 additional imprinted genes have been identified from this domain, including paternally expressed *Usp29*, *Zfp264*, *APeg3* and maternally expressed *Zim1*, *Zim2*, *Zim3* [4,5]. As seen in other imprinted domains, the imprinting and transcription of this imprinted domain is likely regulated through small genomic regions, termed ICRs (Imprinting Control Regions) [6–8]. One genomic region surrounding the promoters of *Peg3* and *Usp29*, termed the Peg3-DMR (Differentially Methylated Region), has been hypothesized to be an ICR for this imprinted domain due to the following features. First, this genomic region has an unusual tandem array of YY1 binding sites [9–11]. Second, the allele-specific DNA methylation on the Peg3-DMR is set up during oogenesis and maintained throughout the lifetime of mammalian species [12–14]. These features are often associated with other ICRs, such as the ICR of *H19*/*Igf2* [11]. A series of subsequent analyses indeed confirmed ICR roles for the Peg3-DMR and also the involvement of *Yy1* in the transcription control and DNA methylation of the *Peg3* domain [15–18]. In particular, the reduced levels of YY1 protein have been shown to up-regulate the expression levels of the *Peg3* domain and also to change the DNA methylation levels of the Peg3-DMR [15–17]. Thus, it has been hypothesized that *Yy1* is a major trans factor regulating the transcription and imprinting of the *Peg3* domain [11].

The protein YY1 is a well-known DNA-binding protein with various functions [19,20]. YY1 can function as a repressor and an activator for the transcriptional regulation of the associated genes [19,20]. YY1 is also known to interact with many protein complexes that are involved in histone modifications [21,22]. According to the recent studies, YY1 interacts with two major epigenetic modifiers, including PRC1 (Polycomb Repressive Complex 1) and KAP1 (KRAB A box-Associated Protein 1)/SETDB1 (histone-lysine N-methyltransferase SETDB1) [23,24], which may provide clues regarding potential roles for YY1 in genomic imprinting. In the case of PRC1, YY1 interacts with PRC1 through YAF2, which might provide a mechanism for permanent and stable repression for the imprinted genes [23]. On the other hand, the interaction between YY1 and KAP1 has been shown to be very specific in ES cells [24]. This cell-type specific interaction might explain the prevalence of YY1 binding sites within the sequences of all the retrotransposons and endogenous retroviruses as well as potential mechanisms for the repression of these DNA elements during early embryogenesis [25,26]. This protein complex, YY1/KAP1/SETDB1, is particularly relevant to genomic imprinting since DNA methylation on several ICRs with YY1 binding sites all occur during early embryogenesis and gametogenesis [11,17]. Nevertheless, it is currently unknown how YY1 is involved in establishing DNA methylation on ICRs and other retrotransposons in mammalian genomes.

In the current study, therefore, we sought to characterize the *in vivo* roles of *Yy1* in the *Peg3* domain using various breeding schemes with a set of newly established mutant alleles of *Peg3*. According to the results, *Yy1* indeed functions as a repressor for the *Peg3* domain. During the course of this study, we have also discovered that *Peg3* is expressed bi-allelically in a small subset of cells in mouse brain.

## Results

### Generation of mutant alleles for *Peg3* and *Yy1*

**Fig 1:**
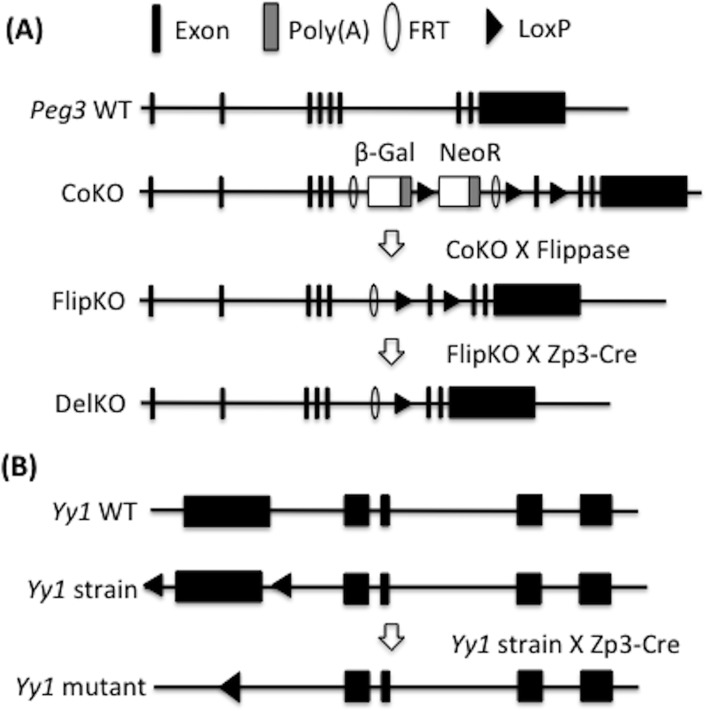
Genomic structures of the wild-type and mutant alleles of *Peg3* and *Yy1*. (**A**) Schematic representation of the wild-type and mutant alleles of the mouse *Peg3* locus. The 9 exons of *Peg3* are indicated by closed boxes in the wild-type (WT) allele. The conditional knockout (CoKO) allele has a 7.1-kb insertion containing a promoterless β-galactosidase (*β-Gal*) and human β-actin promoter-driven neomycin resistant gene (*NeoR*). The insertion cassette in the CoKO allele has been removed through FLP-mediated recombination, producing the FlipKO allele. In the FlipKO allele, two LoxP sites flank the exon 6 of *Peg3*. The Cre recombinase has been used for deleting the exon 6, deriving the DelKO allele for the *Peg3* locus. (**B**) Schematic representation of the wild-type and mutant alleles of the mouse *Yy1* locus. The 1^st^ exon of *Yy1* has been deleted through Cre-mediated recombination, generating the mutant strain for *Yy1*.

The *in vivo* roles of *Yy1* in the *Peg3* domain were investigated using the following mutant alleles, *Peg3* ^*tm1aEUCOMMhmgu*^ and *Yy1* ^*tm2Yshi*^ (**Fig. 1**). First, the *Peg3* locus was initially targeted through inserting an expression cassette carrying a promoterless *LacZ* (β-galactosidase) and *NeoR* (neomycin resistance gene) into its 5^th^ intron [27]. In this knock-in/knock-out scheme, the 3’-side homologous hook contains two LoxP sites flanking the exon 6, deriving a mutant allele that can be ready for conditional knockout experiments. Thus, this mutant allele was named a conditional knockout-ready (CoKO) allele. This CoKO allele was also designed to have immediate mutational effects through truncating the transcription of *Peg3* through two poly(A) signals that had been included as part of the inserted cassette (**Fig. 1A**). The predicted mutational effects have been recently confirmed through a study revealing the complete truncation and subsequent growth-related phenotypes among the mutant mice carrying the CoKO allele [28]. The inserted cassette is also flanked by two FRT sites, and thus the mutational effects by the CoKO allele can be rescued by FLP-mediated recombination, deriving a reverted allele (FlipKO). Finally, the FlipKO allele can be mutated again through the Cre-mediated recombination, resulting in the deletion of the exon 6 (DelKO). Both the FlipKO and DelKO alleles have been successfully generated through two consecutive but separate recombination events, and the mutant strains carrying these two alleles indeed displayed the expected outcomes, the absence and presence of growth-related phenotypes, respectively.

The mutant allele of the *Yy1* locus has been derived from the floxed allele of *Yy1* through Cre-mediated recombination (**Fig. 1B**). This recombination deleted the 3.4-kb genomic region encompassing the promoter and first exon of *Yy1*, abolishing the transcription and translation of *Yy1*. According to the results from initial breeding experiments, the homozygotes carrying the mutant allele were embryonic lethal, while the heterozygotes tend to exhibit smaller body size than their wild-type littermates. This is consistent with the observations derived from previous studies [29]. Interestingly, we have also observed a statistically significant gender ratio among the heterozygotes (male: female = 18: 3) (*X* ^*2*^ test: *X* ^*2*^ = 10.714; df = 1; p = 0.0011) although a small number of litters were tested (S1A **Fig.**). The females are less represented in the heterozygous pool of neonates, which might be caused by *Yy1* effects on the *Xist* locus. Overall, 3 different mutant alleles (CoKO, FlipKO, DelKO) for *Peg3* and one mutant allele (Yy1 mutant) for *Yy1* were successfully generated for a series of breeding experiments as described below.

### Breeding of CoKO and DelKO with *Yy1* mutant strains

**Fig 2:**
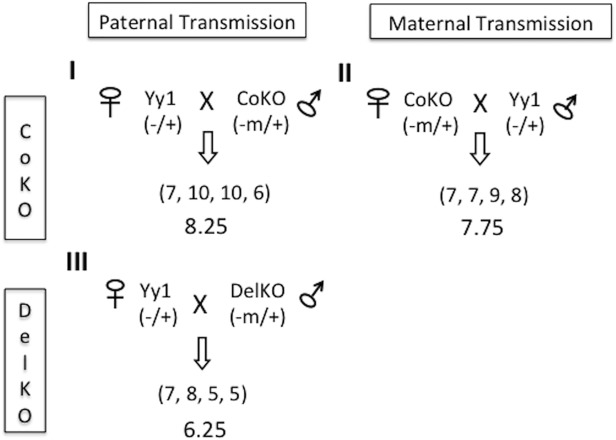
Breeding schemes used to characterize *Yy1* roles in the *Peg3* imprinted domain. This study used three breeding schemes: Breeding I, female heterozygotes for *Yy1* X male heterozygotes for CoKO allele of *Peg3*; Breeding II, female heterozygote for CoKO X male heterozygotes for *Yy1*; and Breeding III, female heterozygotes for *Yy1* X male heterozygotes for DelKO allele of *Peg3*. All of the *Peg3* heterozygotes used for these breeding schemes had inherited the mutant alleles maternally, CoKO (-m/+), and DelKO (-m/+). The average litter sizes of the one-day-old progeny are presented in each breeding setup.

We used the following strategy to test the gene dosage effects of *Yy1* on the *Peg3* domain *in vivo* (**Fig. 2**). This strategy involves the crossing of the mutant alleles of two genetic loci, *Peg3* and *Yy1*, wherein the mutant alleles of *Peg3* serve as a reporter to monitor the gene dosage effects of *Yy1*. The CoKO allele expresses β-galactosidase (β-Gal) under the control of the endogenous promoter of *Peg3* so that potential *Yy1* dosage effects on *Peg3* can be inferred through the activity of β-Gal or RT-PCR utilizing the sequence of β-Gal. Two parental alleles of *Peg3* are also functionally different due to genomic imprinting by the active paternal versus repressed maternal alleles. Thus, *Yy1* gene dosage effects on the paternal and maternal alleles of *Peg3* were analyzed separately through a set of reciprocal breeding schemes (Breeding I and II). In Breeding I and II, the female and male heterozygotes (hets) for the mutant allele of *Yy1* were crossed with the male and female heterozygotes (hets) for the CoKO allele of *Peg3*, respectively. We also used another mutant allele of *Peg3*, DelKO, as an independent reporter allele for this experiment to rule out any artifacts that could originate from the inserted sequence elements within the CoKO allele, such as β-Gal itself and human β-actin promoter-driven *NeoR*. Thus, the female Yy1 hets were crossed with the male DelKO hets (Breeding III).

We have obtained four litters from each of the three breeding schemes, and examined their litter sizes as well as individual health status by measuring their weights (**Table 1** and S1C **Fig.**). The litter sizes of one-day-old mice derived from breeding I, II, and III (8.25, 7.75, and 6.5 respectively) were close to the normal litter size (8) of the same genetic background (129/B6), indicating no embryonic lethality associated with these breeding schemes. These three breeding schemes produced four different genotypes of the progeny: double hets (*Peg3* ^+/-p^ *Yy1* ^-/+^), Peg3 hets (*Peg3* ^+/-p^), Yy1 hets (*Yy1* ^-/+^), and wild-type (WT). According to the results from genotyping, all four different genotypes were represented at the predicted Mendelian ratio (1: 1: 1: 1) among the progeny from the three breeding experiments, confirming that the progeny with each genotype is viable until birth (S1B **Fig.**). This is again consistent with the previous observation, that no embryonic lethality is associated with these breeding schemes. The weight profiles, however, indicated that the double het progeny tend to be smaller and weaker than their littermates (S1C **Fig.**). Furthermore, none of the double hets from breeding III survived past their weaning age, indicating the severity of the combined mutational effects of *Yy1* and *Peg3* on the postnatal survival of individual mice. In summary, the three breeding schemes successfully produced the progeny with all possible combinations of genotypes.

**Table 1:** Summary of breeding results.

Breeding results for experiments														
Breeding setup	*Peg3* (+/-p) *Yy1* (-/+)		*Peg3* (-m/+) *Yy1* (+/-)		*Peg3* (+/-p)		*Peg3* (-m/+)		*Yy1* (-/+)		WT		Number of litters observed	Average litter size (weaned)
	M	F	M	F	M	F	M	F	M	F	M	F		
I	5	3	-	-	6	1	-	-	6	6	3	3	4	8.25
II	-	-	2	7	-	-	3	7	4	3	2	3	4	7.75
III	2	2	-	-	3	5	-	-	3	3	4	2	4	6.25

### *Yy1* gene dosage effects on paternal allele of *Peg3*

**Fig 3:**
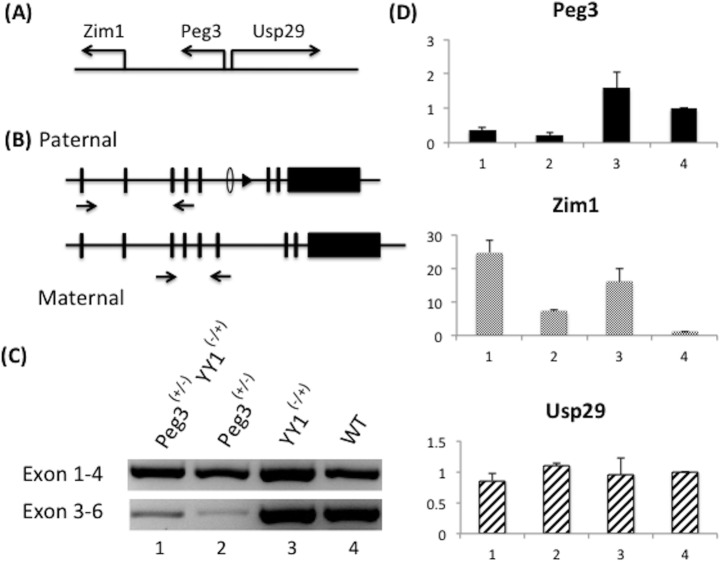
Effects of *Yy1* gene dosage on the *Peg3* imprinted domain. (**A**) Genomic structure of the *Peg3* imprinted domain: maternally expressed *Zim1* and paternally expressed *Peg3* and *Usp29*. (**B**) The current study used two sets of RT-PCR primers for *Peg3*: the first set amplifying exons 1–4 and the second set amplifying exons 3–6. (**C**) RT-PCR analyses of the progeny derived from Breeding I (female Yy1 heterozygotes X male Peg3 DelKO heterozygotes). RT-PCR amplifying exons 1–4 and exons 3–6 were performed using the total RNA isolated from the neonatal brains with 4 genotypes (lanes 1–4). In the case of RT-PCR amplifying exons 1–4, the PCR products from the pups with four genotypes represent the expression from the paternal allele since *Peg3* is paternally expressed. In the case of RT-PCR amplifying exons 3–6, the PCR products from the pups with two genotypes (lane 3, Yy1-/+; lane 4, WT) still represent the expression from the paternal allele of *Peg3*, but the products from the pups with the two other genotypes (lane 1, Yy1-/+ Peg3+/-; lane 2, Peg3+/-) represent the expression from the maternal allele. The mRNA from the paternal allele of *Peg3*, DelKO, cannot be detected by the RT-PCR amplifying exons 3–6 since the exon 6 is deleted in the DelKO allele. (**D**) Quantitative RT-PCR analyses using the total RNA isolated from the pups with four genotypes: Yy1-/+ Peg3+/- (1), Peg3+/- (2), Yy1-/+ (3), WT (4). The expression values of each gene were normalized first with an internal control (*28S*) and later with the values from the WT pup (lane 4). The expression levels of *Peg3* were analyzed using the primer set amplifying exons 1–4, thus representing the expression levels of the paternal allele. This series of qRT-PCR analyses were repeated three independent times from cDNA synthesis to qRT-PCR. Error bars indicate standard deviations for observed triplicates.

The gene dosage effects of *Yy1* on the *Peg3* domain was analyzed mainly with the progeny derived from Breeding III crossing female Yy1 hets and male Peg3 DelKO hets. A set of one-day-old pups with four genotypes (double hets, Peg3 DelKO hets, Yy1 hets, WT) was used for preparing total RNA, cDNA and subsequent qRT-PCR analyses (**Fig. 3**). Actual dosage effects of *Yy1* were tested by comparing the expression levels of a given gene between double hets vs Peg3 DelKO hets (lane 1 vs 2 in **Fig. 3C**) and Yy1 hets vs WT (lane 3 vs 4 in **Fig. 3C**). For the *Peg3* locus, two sets of primers were also used to measure the expression levels, which included the primer set amplifying exon 1 through 4 for the paternal allele and the primer set amplifying exon 3 through 6 for the maternal allele expression (**Fig. 3B**). Since the DelKO allele lacks exon 6, the primer set for exon 3–6 will amplify its corresponding product only from the normal maternal allele that has been inherited from female Yy1 hets. On the other hand, the primer set for exon 1–4 will amplify its product mainly from the paternal allele due to the paternal expression of *Peg3*. This series of expression analyses also included the two adjacent genes of *Peg3*, maternally expressed *Zim1* and paternally expressed *Usp29* (**Fig. 3A**). Other imprinted genes, such as *Zim2*, *Zim3* and *Zfp264*, were not included due to their very low expression levels in neonatal brain [30,31].

According to the results from qRT-PCR, the expression levels of the paternal allele of *Peg3* were 1.5-fold higher in double hets compared to Peg3 DelKO hets (lanes 1 and 2, **Fig. 3D**), and Yy1 hets compared to WT (lanes 3 and 4, **Fig. 3D**). In both sets, the half dosage of *Yy1* coincides with the up-regulation of *Peg3*, suggesting a repressor role for *Yy1* in the paternal allele of *Peg3*. Interestingly, the expression level of *Peg3* is 3-folds lower in double hets compared to Yy1 hets (lanes 1 and 3, **Fig. 3D**), and in Peg3 DelKO hets compared to WT (lanes 2 and 4, **Fig. 3D**). This phenomenon is likely associated with exon 6 deletion in both samples; thus, the observed down-regulation might be caused by the degradation of the *Peg3* mRNA lacking exon 6 and thus the ORF (Open Reading Frame) through the NMD (Non-sense mRNA Decay) pathway [32]. In the case of *Zim1*, the half dosage of *Yy1* also correlates with the up-regulation of *Zim1*, yet the levels of this up-regulation (16 fold) were much higher than those observed from *Peg3* (1.5 fold). By contrast, the half dosage of *Yy1* did not result in any major change in the expression levels of *Usp29*. Overall, this series of expression analyses concluded that the half dosage of *Yy1* coincides with the up-regulation of both *Peg3* and *Zim1*, suggesting a repressor role for *Yy1* for both genes in the *Peg3* imprinted domain. This series of analyses was repeated with 3 technical replicates and 2 biological replicates, and the overall conclusion was reproducible with these independent trials.

### *Yy1* gene dosage effects on the maternal allele of *Peg3*

**Fig 4:**
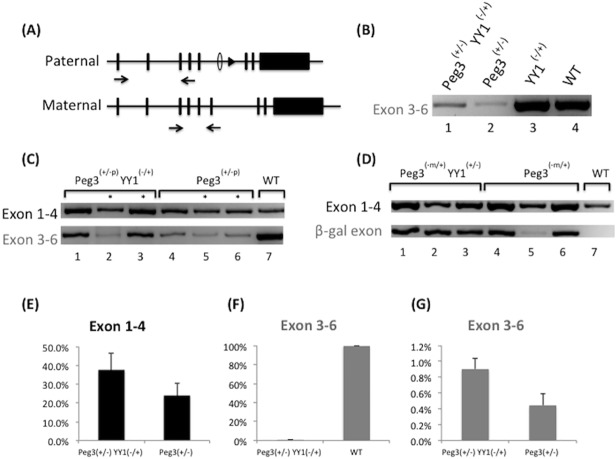
Effects of *Yy1* gene dosage on the maternal expression of *Peg3*. (**A**) Schematic representation of the *Peg3* locus showing the positions of the two sets of primers used for RT-PCR. (**B**) RT-PCR products with the primer set amplifying exons 3–6. Comparison of the expression levels between the neonate brains of pups with the following two genotypes, Yy1-/+; Peg3+/- (lane 1) and Peg3+/- (lane 2), indicate up-regulation of the maternal allele of *Peg3* by the half dosage of *Yy1*. (**C**) Additional RT-PCR analyses further confirming the maternal expression of *Peg3* as well as up-regulation of the maternal expression of *Peg3* by half dosage of *Yy1*. Neonate brains of pups with the genotypes Yy1-/+ Peg3+/- (1), Peg3+/- (4), and WT (7) are the same as the samples shown in **Fig. 4*B*** (2), (3), and (4), respectively. An additional set of pups were obtained from Breeding III (marked with an asterisk *), and subsequently used for the RT-PCR analyses. (**D**) *Yy1* dosage effect on the maternal allele of *Peg3*. Additional RT-PCR analyses confirming the maternal expression of Peg3 with an additional set of pups (1–7) obtained from breeding II (CoKO maternal transmission with a half dosage of Yy1). The primer set amplifying exon 1–4 was used to illustrate the paternal allele expression of *Peg3*, while the primer set amplifying the β-Gal insertion cassette (exon 3 - β-Gal pseudo-exon) was used to show the maternal allele expression of Peg3. (**E**) qRT-PCR analyses measuring the levels of the paternal allele of *Peg3* affected by the half dosage of *Yy1*. This analysis used the primer set amplifying exons 1–4. (**F**) qRT-PCR analysis showing relative expression levels of the maternal to paternal allele of *Peg3*. This analysis used the primer set amplifying exons 3–6. (**G**) qRT-PCR analyses measuring the expression levels of the maternal allele of *Peg3* affected by the half dosage of *Yy1*. This analysis used the primer set amplifying exons 3–6.

*Yy1* dosage effects on the maternal allele of *Peg3* were initially analyzed by detecting the expression of β-Gal in the whole mount and sectioned samples prepared from the progeny of Breeding II inheriting the CoKO allele with β-Gal maternally (**Fig. 2**). Although this series of experiments was not fruitful due to the low sensitivity of the β-Gal staining, we were able to detect low levels of the maternal expression of *Peg3* through RT-PCR (**Fig. 4D**). This suggests that the paternal allele of *Peg3* is intact and functional, and yet the maternal allele, CoKO, is still expressed (**Fig. 4D**). To further investigate the observed maternal expression of the *Peg3* locus, we decided to use the progeny of Breeding III inheriting the DelKO allele paternally, based on the following reason. The maternal allele in this progeny contains the normal, unmodified *Peg3* locus, yet it can be differentiated from the paternal DelKO allele (lacking exon 6) with the primer set amplifying *Peg3* exon 3–6 (**Fig. 4A**). According to the initial survey (**Fig. 4B**), the low levels of *Peg3* expression from the maternal allele were indeed observed from the neonate brains among all progeny with the inherited DelKO allele (**Fig. 4C**). This confirmed the maternal, and thus bi-allelic, expression of the *Peg3* locus in the brain. Subsequent qRT-PCR analyses further revealed that the relative expression level of the maternal allele of *Peg3* was about 0.5% of the paternal allele (**Fig. 4F, G and S3 Fig.**). The half dosage of *Yy1* also coincided with the 1.5-fold up-regulation of *Peg3* (**Fig. 4G**), which is similar to the up-regulation level seen in the paternal allele (**Fig. 4E**). Given the similar changes of *Peg3* expression levels observed between the paternal and maternal alleles by a half dosage of *Yy1*, this is considered to be a transcriptional up-regulation of the already active maternal allele, rather than de-repression of the repressed maternal allele by genomic imprinting (**Fig. 4**). In summary, this series of analyses concluded that the maternal allele of *Peg3* is normally expressed at very low levels in the brain, and that the half dosage of *Yy1* also causes an up-regulation of *Peg3* on the maternal allele.

### Bi-allelic expression of *Peg3* in the specific areas of mouse brain

**Fig 5:**
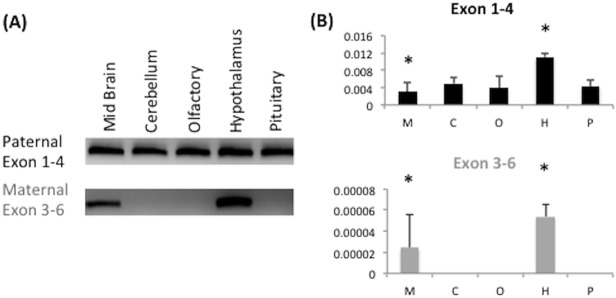
Maternal expression of *Peg3* in mouse brain. (**A**) RT-PCR testing the maternal expression of *Peg3*. The total RNA was isolated from the different parts of a 4-month-old male mouse with the paternally transmitted DelKO allele (midbrain, cerebellum, olfactory, hypothalamus, and pituitary). These RNA were analyzed with the two sets of primers amplifying exon 1–4 and exon 3–6, confirming the maternal expression of *Peg3* in the midbrain and hypothalamus. (**B**) qRT-PCR analyses were also performed to measure the relative expression levels of the paternal and maternal alleles of *Peg3* between the RNA samples isolated from the different parts of the adult brain, including the midbrain (M), cerebellum (C), olfactory (O), hypothalamus (H), and pituitary (P). Parts of the adult mouse brain showing *Peg3* maternal allele expression have been marked with an asterisk (*).

**Fig 6:**
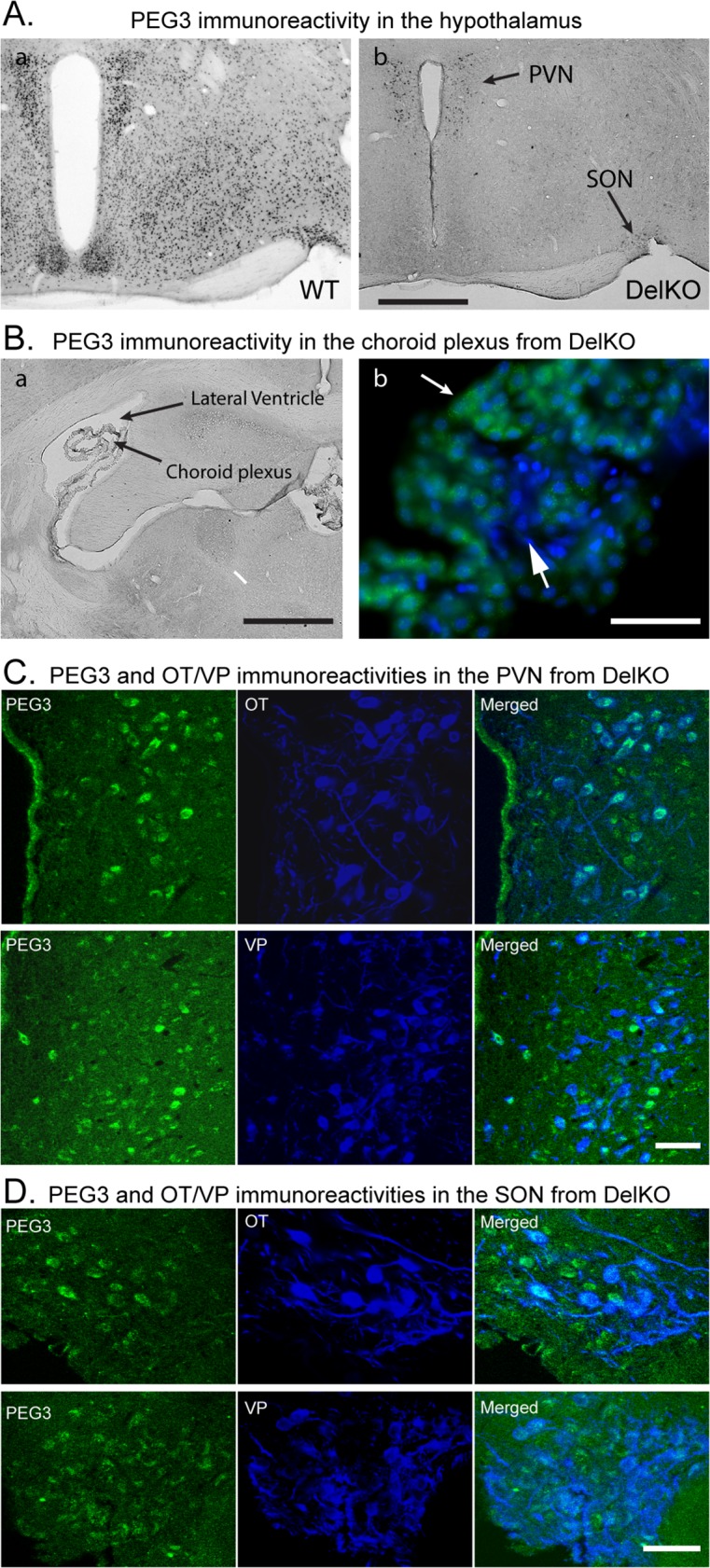
PEG3 immunoreactivity in adult mouse hypothalamus. **(A) PEG3 immunoreactivity in the** adult mouse hypothalamus of two littermates. Image **a** represents global PEG3 immunoreactivity in a 4-month-old, wild-type female mouse brain. Image **b** represents a 4-month-old female mouse brain including the paternally transmitted DelKO allele with arrows indicating PEG3 immunoreactive neurons located in the paraventricular nucleus (PVN) and the supraoptic nucleus (SON). (**B**) PEG3 immunoreactivity of the maternal allele in the choroid plexus from the paternally transmitted DelKO allele. Image **a** represents PEG3 immunoreactivity of the choroid plexus located in the lateral ventricle. Image **b** represents immunoreactivity of PEG3 (green) and DRAQ5 nuclear staining (blue), with a smaller arrow indicating PEG3 immunoreactive cuboidal choroid plexus epithelia, and a larger arrow indicating non-PEG3 immunoreactive endothelia of capillaries. (**C**) Double immunostaining of PEG3 (green) and Oxytocin-OT/Vasopressin-VP (blue) immunoreactive neurons in the PVN from the paternally transmitted DelKO allele. Overlay of PEG3/OT and PEG3/VP indicates PEG3 immunoreacitivity of the maternal allele coinciding predominantly with OT/VP immunoreactive neurons in the PVN. (**D**) Double immunostaining of PEG3 (green) and Oxytocin-OT/Vasopressin-VP (blue) immunoreactive neurons in the SON from the paternally transmitted DelKO allele. Overlay of PEG3/OT and PEG3/VP indicates PEG3 immunoreacitivity of the maternal allele coinciding predominantly with OT/VP immunoreactive neurons in the SON.

The observed low levels of expression from the maternal allele of *Peg3* were further investigated with RT-PCR (**Fig. 5**) and immunohistochemistry (**Fig. 6**). We first surveyed the maternal expression of *Peg3* using the total RNA isolated from the neonate mouse heads of the Peg3 het mice inheriting the DelKO allele paternally (**Fig. 4B and C**). We repeated an RT-PCR assay on a set of the total RNA isolated from the different parts of the adult mouse brain (**Fig. 5A**). The maternal expression was detected mainly in the hypothalamus and mid brain sections with the expression levels being slightly higher in the hypothalamus than in the mid brain (**Fig. 5B**). This was somewhat different from the expression pattern observed from the paternal allele, which showed global expression throughout the entire brain. This suggests that the observed maternal expression is specific to certain areas of the brain including the midbrain and the hypothalamus regions. The relative expression levels of maternal to paternal alleles in these cell types are much lower based on qRT-PCR data (about 1% of the paternal level, **Fig. 5B and S3 Fig.**). This suggests that *Peg3* expression is most likely bi-allelic in a small population of cells in the midbrain and hypothalamus regions. Moreover, DNA methylation analyses using DNA derived from tissues pertaining to bi-allelic expression shows no major methylation differences when compared to tissues derived from other areas of the brain (S2 **Fig.**).

To elucidate the location of maternal *Peg3* expression in the brain, we performed immunostaining of PEG3 using a series of whole coronal sections containing the hypothalamus (10 sections collected every 200 μm) from an adult Peg3 hets inheriting the CoKO and DelKO alleles paternally (**Fig. 6**). Brain slices from the wild-type littermates were also included as a positive control. There are numerous PEG3 immunoreactive cells ubiquitously located in the brain sections from WT mice. The intensity of immunoreactivity appeared especially high in the hypothalamic region (**Fig. 6Aa**) and in the choroid plexus in the lateral ventricles (**Fig. 6Ba**). In contrast, there was no detectable PEG3 immunoreactivity in the brain slices from the paternally transmitted CoKO or DelKO mice, except in the hypothalamic paraventricular (PVN) and supraoptic (SON) nuclei (**Fig. 6Ab**) and in the choroid plexus (**Fig. 6Ba**), which was expected given the very low (0.5%) maternal expression of *Peg3*. Prominent PEG3-immunoreactive cells observed in the PVN and SON in the CoKO and (of) DelKO mice (**Fig. 6Ab)** is also consistent with the results from RT-PCR, which showed *Peg3* expression in the hypothalamus (**Fig. 5A and B**). The choroid plexus is a tuft of capillaries with an overlaying epithelial covering. PEG3 immunofluorescence labeling was counterstained with a DNA dye, DRAQ5, to investigate whether PEG3 immunoreactivity is located in the capillary lining enodothelial cells or the choroid plexus epithelial cells. PEG3 immunoreactivity was located exclusively in the cuboidal epithelia typically found in the choroid plexus epithelium, but was absent from the endothelia of the capillaries (**Fig. 6Bb**). Because the PVN and SON contain neurons synthesizing neurohypophysial hormones, oxytocin and vasopressin, double immunofluorescence detection of PEG3 and oxytocin/vasopressin was employed to determine whether the maternal expression of *Peg3* occurs specifically in oxytocin-and/or vasopressin-synthesizing neurons. The double labeling revealed that PEG3-immunoreactivity was found in oxytocin- and vasopressin- immunoreactive neurons in both the PVN (**Fig. 6C**) and SON (**Fig. 6D**). In summary, this series of qRT-PCR and immunostaining experiments strongly suggest the maternal allele expression of *Peg3* in specific cell types of the mouse brain, thus confirming its bi-allelic expression in these cell types.

## Discussion

In the current study, we tested the *in vivo* effects of *Yy1* gene dosage on the *Peg3* imprinted domain with various breeding schemes utilizing the mutant alleles. The results indicated that the half dosage of *Yy1* coincides with the up-regulation of *Peg3* and *Zim1*, suggesting a repressor role of *Yy1* in the imprinted domain. The results also posed an unexpected observation that the maternal allele of *Peg3* is normally expressed, and thus the expression of *Peg3* is bi-allelic in the specific areas of the brain, such as the choroid plexus and the SON and PVN of the hypothalamus. Overall, these results confirm the *in vivo* repressor role of *Yy1* that had been previously observed from *in vitro* studies, and also provide a new insight regarding the bi-allelic expression of *Peg3* in the mouse brain.

According to the present study results (**Fig. 3**), the half dosage of *Yy1* appears to coincide with the up-regulation of *Peg3* and *Zim1*, suggesting a repressor role for *Yy1* in the *Peg3* domain. A similar observation has been previously made multiple times through a series of *in vitro* and *in vivo* experiments, demonstrating the global up-regulation of the *Peg3* domain in a response to the low levels of the YY1 protein [15–17]. This domain-wide response along with multiple YY1 binding sites within the 1^st^ intron of *Peg3* have been the two major observations suggesting the possibility that *Yy1* is a major trans factor controlling the transcription of this 500-kb domain [11]. This prediction is overall well supported by the current study utilizing much more controlled *in vivo* systems than the previous *in vitro* systems [15–17]. Nevertheless, the current study was unable to replicate another previous observation that the low levels of YY1 protein may be responsible for DNA hypomethylation on the Peg3-DMR (S2 **Fig.**). According to the results (**Fig. 4**), the half dosage of *Yy1* does not appear to affect the epigenetic imprint of the maternal allele of *Peg3* although this is still somewhat inconclusive due to the technical limitations associated with the sensitivity of the β-Gal staining. Instead, the boosted expression levels observed from the maternal allele of *Peg3* in Yy1 hets compared to those of WT mice is thought to be caused by the up-regulation of the already active maternal allele, rather than by the de-repression of the repressed maternal allele via DNA hypomethylation. Although we need to further investigate this aspect in the near future, the inability to detect the predicted hypomethylation in the current study could be due to the following reasons. First, half dosage of *Yy1* might not be sufficient enough to derive a similar observation made from *in vitro* studies. Second, the pups severely affected by the DNA hypomethylation might not be viable so that the breeding schemes used for the current study could not produce the pups with predicted epigenetic imprints. Overall, the current study utilizing *in vivo* systems again confirms that *Yy1* functions as a transcriptional repressor for the *Peg3* imprinted domain.

Given the fact that one allele of *Peg3* is already repressed by genomic imprinting, it is interesting to speculate why the remaining active allele requires further repression by another transcription factor, *Yy1*. This may be related to the potential functions of *Peg3*. According to the recent studies, *Peg3* is predicted to be a major regulator controlling autophagy in endothelial cells [33,34]. Many stimuli from environment, such as starvation, can stimulate autophagy along with *Peg3*, resulting in a temporal up-regulation of *Peg3*. Restoring it back to normal levels of *Peg3* is likely required for the proper function of cells, which may use other unknown regulatory mechanisms. In that regard, it is relevant to note that the mTOR (mechanistic target of rapamycin) signaling pathway is known to repress autophagy, in which *Yy1* acts as a major contributing factor [35]. Thus, it is reasonable to predict that *Yy1* may be involved in controlling the dynamically fluctuating levels of *Peg3*, which may be triggered by environmental and developmental cues. Recent studies on histone modification profiles have also indicated that promoters of several imprinted genes in the *Peg3* domain interacts with one evolutionarily conserved region, ECR18, suggesting its key roles played in the transcription and imprinting control of *Peg3* domain as a distant regulatory element [36]. Thus, it is conceivable that *Yy1* may affect histone modification profiles contributing to the up-regulation of *Peg3* expression instead of DNA methylation changes, using such distant regulatory elements. Although speculative, this may be a reason why *Yy1* is needed for the repression of *Peg3*, which requires further investigation in near future.

The expression of *Peg3* appears to be bi-allelic in the specific areas of the brain (**Figs. 5 and 6**). The detection of the maternal expression of *Peg3* from the both mutant models, CoKO and DelKO, rules out the possibility that this detection is due to unknown artifacts associated with mutagenesis. Also, this rules out the possibility that the observed maternal expression of *Peg3* is caused by some functional compensation between two parental alleles. For instance, the loss-of-function type mutation on the paternal allele (DelKO) might render the cells to de-repress the repressed maternal allele. In the case of the progeny inheriting the CoKO allele maternally (**Fig. 2**), however, the paternal allele of *Peg3* is intact and functional, and yet the maternal allele, CoKO, is still expressed (**Fig. 4D**). Therefore, this strongly supports the idea that the observed maternal expression is reflecting the genuine bi-allelic expression of *Peg3* in normal mice, which has been previously unnoticed. Nevertheless, this new observation is intriguing given the following reasons. First, there is another imprinted gene, *Igf2*, which is known to be bi-allelic in the choroid plexus [37,38]. Given a very small number of imprinted genes in mammalian genomes, the bi-allelic expression of two imprinted genes (*Peg3* and *Igf2*) in the same small area of mouse brain seems to be a very rare coincidence. At the same time, the choroid plexus is known to play a major role in controlling the concentration of ions such as Na^+^, Cl^-^, HCO_3_^-^, and K^+^ in the cerebrospinal fluid, and thus should be very critical for the normal function of neurons in the brain. Thus, this rare coincidence may be an indication that some functional constraints derive the bi-allelic expression of these two imprinted genes in the choroid plexus. Second, several previous reports have predicted that the maternal allele of *Peg3* may be functional at some unknown stages and/or in specific cell types since the homozygous animals for several mutant alleles targeting the *Peg3* locus are not viable although the paternal heterozygotes are still viable [18,28]. This prediction is further supported by the observed bi-allelic expression of *Peg3* in that the maternal allele of *Peg3* is indeed expressed and functional in the specific areas of brains. The lack of both the paternal and maternal expression of *Peg3* in these brain areas might contribute to the observed lethality of the homozygous mutant animals. Although this is likely, we need to first investigate the functional contribution of the maternal allele of *Peg3* to the choroid plexus and other areas, such as the PVN and SON of hypothalamus. Overall, the current study reports, for the first time, the bi-allelic expression of *Peg3* in specific areas of mouse brain, and thus it would be of great interest to follow up the functional significance of the observed bi-allelic expression in the near future.

## Materials and Methods

### Ethics Statement

All the experiments related to mice were performed in accordance with National Institutes of Health guidelines for care and use of animals, and also approved by the Louisiana State University Institutional Animal Care and Use Committee (IACUC), protocol #13-061.

### Generating the mutant strains for *Yy1* and *Peg3*

The current study used the following 7 mouse strains. The strain carrying a floxed allele for *Yy1* was obtained from the Jackson Lab (Stock No. 014649, B6.129S4-*Yy1* ^*tm2Yshi*^/J) [29]. The strain for the CoKO allele of *Peg3* was made using a targeted ES cell from the EUCOMM (European Conditional Mouse Mutagenesis program), and this strain has been maintained in the lab [28]. These two strains were crossed with the Zp3-Cre line from the Jackson Lab (Stock No. 003651, C57BL/6-Tg (Zp3-cre) 93Knw/J) and the Rosa26-FLP line from Jackson Lab (Stock No. 009086, B6.129S4-*Gt* (ROSA)*26Sor* ^*tm1(FLP1)Dym*^/RainJ). The mutagenesis through these breeding derived the *Yy1* mutant strain, and also the FlipKO and DelKO strains for the *Peg3* locus. The following primer sets were used for genotyping of these strains: the deletion of exon 1 for *Yy1*, YY1-CoKO-F (5’-ACCTGGTCTATCGAAAGGAAGCAC-3’) and YY1-genotype-R (5’-TCATCCAAAGTTCGAAACCTGCTTTCC-3’); the presence of the expression cassette for the CoKO allele, Peg3-5arm (5’-CCCTCAGCAGAGCTGTTTCCTGCC-3’) and LAR3 (5’-CAACGGGTTCTTCTGTTAGTCC-3); the deletion and detection of the expression cassette for FlipKO and DelKO, respectively, Peg3-CoKO-F (5’-ACCTGGTCTATCGAAAGGAAGCAC-3’) and LoxR (5’-TCATCCAAAGTTCGAAACCTGCTTTCC-3’); the presence of Zp3-Cre, Zp3-cre-F (5’-TAGGAATCACGTGGAGTGTCT-3’) and oIMR1085 (5’-GTGAAACAGCATTGCTGTCACTT-3’); the presence of Rosa26-FLP, oIMR0853 (5’-GCGAAGAGTTTGTCCTCAACC-3’) and oIMR0852 (5’-AAAGTCGCTCTGAGTTGTTAT-3’). DNA was isolated from ear or tail snipes through incubating the tissues at 65°C with the tail lysis buffer (50 mM Tris-Cl at pH 8.0, 100 mM EDTA at pH 8.0, 250 mM NaCl, 1% SDS, along with 20 μg/mL Proteinase K). PCR premix kit (Intron Biotech) was used for genotyping at the following conditions (step 1, 95°C-30 sec; step 2, 95°C-30 sec, 60°C-30 sec, 72°C-60 sec for 33 cycles; step 3, 72°C-7 min). The information regarding individual primer sequences are also available (S1 **Table**).

### Breeding experiments

The current study used the following three breeding schemes: Breeding I, female heterozygotes for *Yy1* X male heterozygotes for CoKO of *Peg3*; Breeding II, female heterozygotes for CoKO X male heterozygotes for *Yy1*; Breeding III, female heterozygotes for *Yy1* X male heterozygotes for DelKO (**Fig. 2**). The health status of the pups from these breeding was monitored through measuring their birth weight (S1C **Fig.**). The gender of these pups was also determined through PCR with the following primer set: mSry-F (5’-GTCCCGTGGTGAGAGGCACAAG-3’) and mSry-R (5’-GCAGCTCTACTCCAGTCTTGCC-3’). All animals were kept in a temperature-controlled environment at 22°C, with 4–5 mice per cage over a 12-hour period of light/dark cycles. Litter size, genotype, birth weight and gender were all recorded for each mating pair, which were later used to generate a graphical representation of the gender and genotype distribution for each cross (S1A-B **Fig.**).

### RNA isolation and quantitative RT-PCR analysis

Total RNA was isolated from the brains of one-day-old neonates using a commercial kit (Trizol, Invitrogen) according to the manufacturer’s protocol. The total RNA was then reverse-transcribed using the M-MLV kit (Invitrogen), and the subsequent cDNA was used as a template for quantitative PCR. This analysis was performed with SYBR Select Master Mix (Applied Biosystems, Life Technologies) using the iCycler iQTM multicolor real-time detection system (Bio-Rad). All qRT-PCR reactions were carried out for 40 cycles under standard PCR conditions with internal controls (*28S* and *β-actin*). The results derived from qRT-PCR were further analyzed using the threshold (Ct) value. The experiments were performed in triplicates for each imprinted gene (*Peg3*, *Zim1*, *Usp29*). The ΔCt value was initially calculated by subtracting Ct value of a testing replicate of a given gene from the average Ct value of the internal control (*28S* and *β-actin*). The fold difference for each replicate was then calculated by raising the ΔΔCt value as a power of 2 [39]. The relative expression levels of all samples were then calculated by dividing the calculated expression level of each sample by the expression level of the wild-type sample. The average and standard deviation for each sample were then calculated by compiling the normalized values. The information regarding individual primer sequences are also available (S2 **Table**).

### Immunohistochemistry

Mice were deeply anesthetized with Nembutal (50mg/kg; i.p) and intracardially perfused with 0.1 M sodium phosphate-buffered saline (PBS: pH 7.2–7.4) followed by a fixative of 4% paraformaldehyde in 0.1 M phosphate buffer (PB: pH 7.2–7.4). Brains were dissected and post-fixed in the same fixative overnight, then transferred to 30% sucrose in 0.1 M PB overnight. Coronal sections (40-μm) containing the SON and PVN were obtained by a sliding microtome (Leica SM2010R, Leica, Mannheim, Germany). Polyclonal antibody against PEG3 was raised in rabbit and used in free-floating brain slices overnight at 4°C at a concentration of 1:2,000 [16] in PBS containing 0.5% Triton X-100 (PBST). For single staining of PEG3, the brain slices were subsequently incubated with biotinylated goat anti-rabbit antibody (Vector Labs, Burlingame, CA) at 1:200 in PBST. Antigen-antibody interaction was visualized by the ABC-diaminobenzidine method according to the protocol provided by Vector Labs (Burlingame, CA). The brain sections were mounted on gelatin-coated slides, dehydrated, cleared and cover slipped with Permount. The brain sections were rinsed 3 times for 5 min with PBST between each step.

For double immunofluorescence, the brain sections were incubated with PEG3 antibody, followed by incubation with goat anti-rabbit antibody conjugated with DyLight 488 (Jackson ImmunoResearch, West Grove, PA) at 1:400 in PBST overnight. Subsequently, sections were incubated with either oxytocin-neurophysin (NP) or vasopressin-NP mouse monoclonal antibodies (PS38 and PS41, respectively: provided by H. Gainer, NIH) at 1:500 in PBST overnight, followed by incubation with goat anti-mouse antibody conjugated with DyLight 649 (Jackson ImmunoResearch, West Grove, PA) at 1:400 in PBST overnight. In some cases, brain sections were counter stained with DNA labeling dye, 1,5-bis{(2-(di-methylamino)ethyl)amino}-4, 8-dihydroxyanthracene-9,10-dione (DEAQ5), according to manufacture’s instructions (BioStatus limited, Thermo Scientific). The sections were mounted in polyvinyl alcohol (PVA) with anti-fading agent 1,4-diazabicyclo(2.2.2)octane (DABCO) that consists of 4.8g PVA, 12g glycerol, 12mL dH_2_O, 24 mL0.2M Tris-HCl, and 1.25g DABCO. Images were acquired with a confocal microscope Leica TCS SP2 spectral confocal microscope, Mannheim, Germany). Optical section thickness was 1 μm. These were viewed in stacks of 5 sections using ImageJ software (NIH).

## Supporting Information

S1 Fig Genotype and weight profiles of the breeding schemes. **(A)** Yy1 strain breeding results. Representation of the male and female Yy1 hets and WT, observed in 6 litters of adult mice. There was no significant difference observed between Yy1 hets versus WT offspring (*X* ^*2*^ test: *X* ^*2*^ = 0.091; df = 1; p = 0.7630), although a significant difference was observed between males versus females in Yy1 hets (*X* ^*2*^ test: *X* ^*2*^ = 10.714; df = 1; p = 0.0011). **(B)** Graphical representation of the genotype distribution (double het, Peg3 het, Yy1 het, and WT) for breeding I, II, and III corresponding to CoKO paternal transmission, CoKO maternal transmission and DelKO paternal transmission with Yy1 het, respectively. A total of 4 litters were used for this analysis consisting of approximately 31 individuals for CoKO paternal transmission, 33 individuals for CoKO maternal transmission, and 23 individuals for DelKO paternal transmission. **(C)** A graphical representation of the weight distribution for all four genotypes observed from the breeding schemes representing CoKO paternal transmission (blue), CoKO maternal transmission (pink), and DelKO paternal transmission (purple). The percentage of birth weight for neonate mice was calculated by comparing the individual weight at birth to the average weight of each litter for a total of 4 litters. The error bars indicate the standard deviation observed between the birth weight percentages among each genotype. CoKO paternal transmission weight comparison between double heterozygous and wild-type neonates indicate a significant difference p = 0.0121 using the student t-test. CoKO maternal transmission weight comparison between double heterozygous and wildtype neonates indicate a significant difference p = 0.0094 using the student t-test. DelKO paternal transmission neonate weight comparison between double heterozygous and wild-type indicates no significant difference showing p = 0.2595 using the student t-test. All two tailed p-values have been calculated using the paired t-test.
(TIF)

S2 Fig DNA Methylation analysis of *Peg3*. Methylation levels of the Peg3-DMR were determined using COBRA. A set of genomic DNA isolated from the cortex and choroid plexus of two mice (WT and Yy1^-/+^) was treated with bisulfite conversion. The amplified PCR products from the Peg3-DMR were digested with *Hph*I and *Taq*Ia enzymes. The digestion pattern revealed half methylation in both CP and CTRL without any major difference, indicating no obvious methylation difference in the choroid plexus with Peg3 biallelic expression. This suggests that small populations of cells are likely bialleleic and/or an unknown alterative promoter may derive the maternal expression. The observed pattern is also true between WT and YY1^-/+^, indicating no major effect on the biallelic expression of Peg3 by Yy1.
(TIF)

S3 Fig Maternal allele expressions of *Peg3* using a mouse hybrid cross. (**A**) Schematic representation of the *Peg3* locus. Positions are indicated for two sets of primers used for qRT-PCR to distinguish the maternal and the paternal alleles of a PWD/B6 hybrid mouse strain. A female PWD mouse was mated with a B6 male to give rise to hybrid progeny. Using two single nucleotide polymorphisms (SNPs), two primers were designed to distinguish *Peg3* alleles from PWD (maternal) and the B6 (paternal). RNA was isolated and subsequent cDNA was generated from the hypothalamus and the rest of the brain from PWD/B6 hybrid progeny to detect allele specific *Peg3* expression levels. (**B**) qRT-PCR analyses measuring the levels of *Peg3* maternal allele expression in PWD/B6 hypothalamus and brain compared to their parental strains. Allele specific reverse primers were combined with a forward primer corresponding to *Peg3* exon 6 to amplify mRNA from *Peg3* exon 6–9 to determine the relative expression levels of *Peg3* in hybrid tissues compared to their parental strains. The average expression levels of *Peg3* was normalized to β-actin and subsequently compared to B6 and PWD respectively. The percentage of maternal *Peg3* expression was calculated using the maternal to paternal expression ratio of the PWD/B6 hybrid tissues.
(TIF)

S1 Table Primer sets used for genotyping. (PDF)

S2 Table Primer sets used for RT-PCR and qRT-PCR experiments. (PDF)
