# Supplementary figures and images for: Yy1 Gene Dosage Effect and Bi-Allelic Expression of Peg3
Source: PLoS One. 2015 Mar 16;10(3):e0119493. doi: 10.1371/journal.pone.0119493 (PMC4361396; doi:10.1371/journal.pone.0119493)

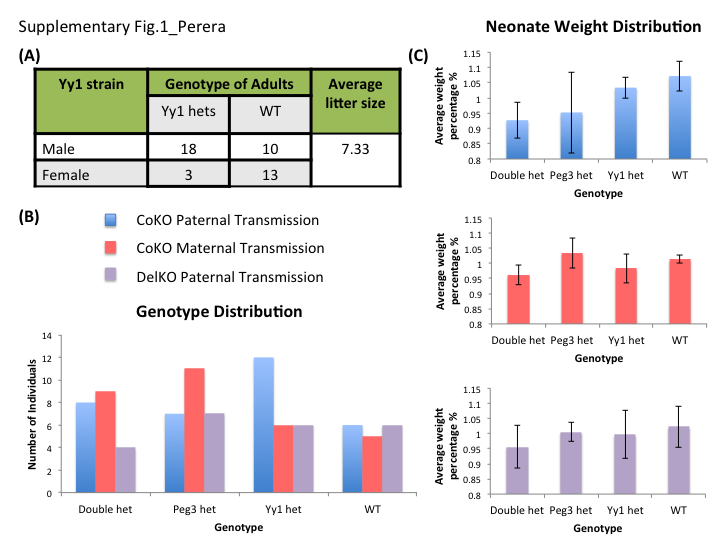

Supplement: S1 Fig — (A) Yy1 strain breeding results. Representation of the male and female Yy1 hets and WT, observed in 6 litters of adult mice. There was no significant difference observed between Yy1 hets versus WT offspring (X 2 test: X 2 = 0.091; df = 1; p = 0.7630), although a significant difference was observed between males versus females in Yy1 hets (X 2 test: X 2 = 10.714; df = 1; p = 0.0011). (B) Graphical representation of the genotype distribution (double het, Peg3 het, Yy1 het, and WT) for breeding I, II, and III corresponding to CoKO paternal transmission, CoKO maternal transmission and DelKO paternal transmission with Yy1 het, respectively. A total of 4 litters were used for this analysis consisting of approximately 31 individuals for CoKO paternal transmission, 33 individuals for CoKO maternal transmission, and 23 individuals for DelKO paternal transmission. (C) A graphical representation of the weight distribution for all four genotypes observed from the breeding schemes representing CoKO paternal transmission (blue), CoKO maternal transmission (pink), and DelKO paternal transmission (purple). The percentage of birth weight for neonate mice was calculated by comparing the individual weight at birth to the average weight of each litter for a total of 4 litters. The error bars indicate the standard deviation observed between the birth weight percentages among each genotype. CoKO paternal transmission weight comparison between double heterozygous and wild-type neonates indicate a significant difference p = 0.0121 using the student t-test. CoKO maternal transmission weight comparison between double heterozygous and wildtype neonates indicate a significant difference p = 0.0094 using the student t-test. DelKO paternal transmission neonate weight comparison between double heterozygous and wild-type indicates no significant difference showing p = 0.2595 using the student t-test. All two tailed p-values have been calculated using the paired t-test. (TIF) [file pone.0119493.s001.tif]

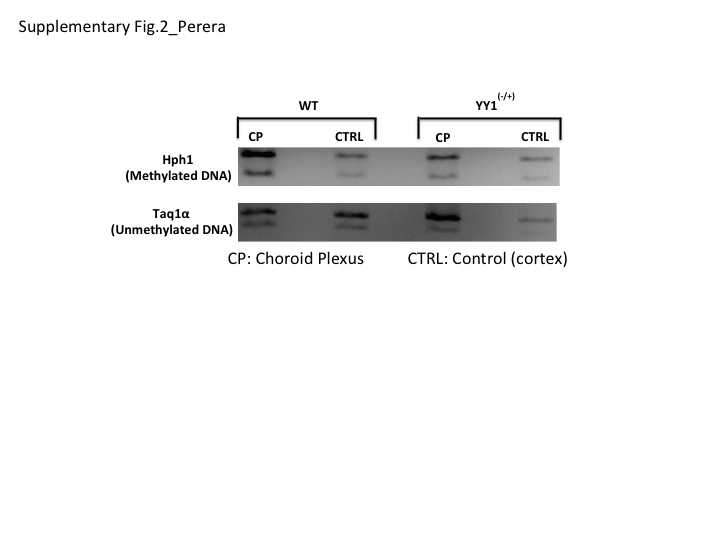

Supplement: S2 Fig — Methylation levels of the Peg3-DMR were determined using COBRA. A set of genomic DNA isolated from the cortex and choroid plexus of two mice (WT and Yy1-/+) was treated with bisulfite conversion. The amplified PCR products from the Peg3-DMR were digested with HphI and TaqIa enzymes. The digestion pattern revealed half methylation in both CP and CTRL without any major difference, indicating no obvious methylation difference in the choroid plexus with Peg3 biallelic expression. This suggests that small populations of cells are likely bialleleic and/or an unknown alterative promoter may derive the maternal expression. The observed pattern is also true between WT and YY1-/+, indicating no major effect on the biallelic expression of Peg3 by Yy1. (TIF) [file pone.0119493.s002.tif]

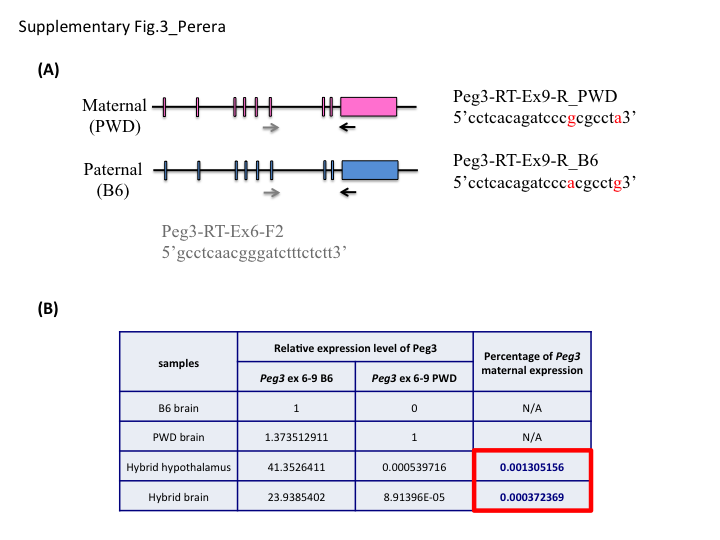

Supplement: S3 Fig — (A) Schematic representation of the Peg3 locus. Positions are indicated for two sets of primers used for qRT-PCR to distinguish the maternal and the paternal alleles of a PWD/B6 hybrid mouse strain. A female PWD mouse was mated with a B6 male to give rise to hybrid progeny. Using two single nucleotide polymorphisms (SNPs), two primers were designed to distinguish Peg3 alleles from PWD (maternal) and the B6 (paternal). RNA was isolated and subsequent cDNA was generated from the hypothalamus and the rest of the brain from PWD/B6 hybrid progeny to detect allele specific Peg3 expression levels. (B) qRT-PCR analyses measuring the levels of Peg3 maternal allele expression in PWD/B6 hypothalamus and brain compared to their parental strains. Allele specific reverse primers were combined with a forward primer corresponding to Peg3 exon 6 to amplify mRNA from Peg3 exon 6–9 to determine the relative expression levels of Peg3 in hybrid tissues compared to their parental strains. The average expression levels of Peg3 was normalized to β-actin and subsequently compared to B6 and PWD respectively. The percentage of maternal Peg3 expression was calculated using the maternal to paternal expression ratio of the PWD/B6 hybrid tissues. (TIF) [file pone.0119493.s003.tif]
