# Supplementary material for: Yy1 Gene Dosage Effect and Bi-Allelic Expression of Peg3
Source: PLoS One. 2015 Mar 16;10(3):e0119493. doi: 10.1371/journal.pone.0119493 (PMC4361396; doi:10.1371/journal.pone.0119493)
Supplement: S1 Table — (PDF) [file pone.0119493.s004.pdf]

**Supplementary Table 1. Primer sets used for genotyping experiments.**

| Locus | Name | Sequence (5' -> 3') | Primer set | Size (bp) | *Position (mm9, NCBI Build 37) |
|---|---|---|---|---|---|
| YY1(Exon1 del) | YY1 CoKO F<br>YY1 genotype R | ACCTGGTCTATCGAAAGGAAGCAC<br>TCATCCAAAGTTCGAAACCTGCTTTCC | 1st primer | ≅290 | Chr12:108,791,900-108,795,566 |
| Zp3-cre | Zp3 F<br>oIMR1085 | TAGGAATCACGTGGAGTGTCT<br>GTGAAACAGCATTGCTGTCACTT | 1st primer | 500 | Chr5:135,979,920-unknown |
| CoKO | Peg3-5 arm<br>Peg3-Lar3 | CCCTCAGCAGAGCTGTTTCCTGCC<br>CAACGGGTTCTTCTGTTAGTCC | 1st primer | ≅510 | chr7:6,630,678-6,634,867 |
| DelKO | Peg3-5 arm<br>Peg3-LoxR | CCCTCAGCAGAGCTGTTTCCTGCC<br>TGAACTGATGGCGAGCTCAGACC | 1st primer | ≅505 | chr7:6,904,937-6,912,835 |
| Y chromosome | mSry-F<br>mSry-R | GTCCCGTGGTGAGAGGCACAAG<br>GCAGCTCTACTCCAGTCTTGCC | 1st primer | 318 | chrY:1,919,207-1,919,524 |
| Flippase | oIMR0853-F<br>oIMR0852-R | GCGAAGAGTTTGTCCTCAACC<br>AAAGTCGCTCTGAGTTGTTAT | 1st primer | ≅330 | Chr6:113,076,214-unknown |
