# Supplementary material for: Yy1 Gene Dosage Effect and Bi-Allelic Expression of Peg3
Source: PLoS One. 2015 Mar 16;10(3):e0119493. doi: 10.1371/journal.pone.0119493 (PMC4361396; doi:10.1371/journal.pone.0119493)
Supplement: S2 Table — (PDF) [file pone.0119493.s005.pdf]

Supplementary Table 2. Primer sets used for RT-PCR and qRT-PCR experiments.

Locus	Name	Sequence (5' -> 3')	Primer set	Size (bp)	*Position (mm9, NCBI Build 37)
Peg3 3-6	Peg3-RT-exon3-F2<br>Peg3-RT-126-2	ATCCCTGAAACGCTCAAGCCCT<br>AAGATCCCGTTGAGGCAGCC	1st primer	291(289)	chr7:6,668,849-6,671,234
Peg3 1-4	Peg3-RT-1a<br>Peg3-RT-124	GGTTCAGTGTGGGTGCACTAGACT<br>TGTCACTGTTGGTGTCGTCT	1st primer	304(301)	chr7:6,670,582-6,683,038
Beta gal	Peg3-RT-exon3-F2<br>Peg3-RT-βgal-pseudo	ATCCCTGAAACGCTCAAGCCCT<br>GAAGGCTGTGCGAGGCCGCTTG	1st primer	≅310	chr7:6,668,849-6,671,234
Zim1	Zim1-RT-c<br>Zim1-RT-d	GATCACCAGGTTGGAGCAAGGAGT<br>AGCGCTCTGTGGTGTTGTAGTTG	1st primer	405	chr7:6,630,678-6,634,871
Usp29	Usp29-RT-PolA<br>Usp29-RT-PolB	GAGCCTGCAGCCGGACCGT<br>GCTCCGGGGCTTCTGCTCT	1st primer	233	chr7:6,904,937-6,912,835
Actin (beta)	bActin-1a<br>bActin-1b	GAGCACCCTGTGCTGCTCACCGA<br>CTCTTTGATGTCACGCACGATTTC	1st primer	344	chr5:143,666,183-143,666,981
